# Molecular basis for defect in Alix-binding by alternatively spliced isoform of ALG-2 (ALG-2^ΔGF122^) and structural roles of F122 in target recognition

**DOI:** 10.1186/1472-6807-10-25

**Published:** 2010-08-06

**Authors:** Tatsutoshi Inuzuka, Hironori Suzuki, Masato Kawasaki, Hideki Shibata, Soichi Wakatsuki, Masatoshi Maki

**Affiliations:** 1 Department of Applied Molecular Biosciences, Graduate School of Bioagricultural Sciences, Nagoya University, Nagoya 464-8601, Japan; 2 Structural Biology Research Center, Photon Factory, Institute of Materials Structure Science, High Energy Accelerator Research Organization (KEK), Tsukuba, Ibaraki 305-0801, Japan

## Abstract

**Background:**

ALG-2 (a gene product of *PDCD6*) belongs to the penta-EF-hand (PEF) protein family and Ca^2+^-dependently interacts with various intracellular proteins including mammalian Alix, an adaptor protein in the ESCRT system. Our previous X-ray crystal structural analyses revealed that binding of Ca^2+ ^to EF3 enables the side chain of R125 to move enough to make a primary hydrophobic pocket (Pocket 1) accessible to a short fragment of Alix. The side chain of F122, facing a secondary hydrophobic pocket (Pocket 2), interacts with the Alix peptide. An alternatively spliced shorter isoform, designated ALG-2^ΔGF122^, lacks Gly^121^Phe^122 ^and does not bind Alix, but the structural basis of the incompetence has remained to be elucidated.

**Results:**

We solved the X-ray crystal structure of the PEF domain of ALG-2^ΔGF122 ^in the Ca^2+^-bound form and compared it with that of ALG-2. Deletion of the two residues shortened α-helix 5 (α5) and changed the configuration of the R125 side chain so that it partially blocked Pocket 1. A wall created by the main chain of 121-GFG-123 and facing the two pockets was destroyed. Surprisingly, however, substitution of F122 with Ala or Gly, but not with Trp, increased the Alix-binding capacity in binding assays. The F122 substitutions exhibited different effects on binding of ALG-2 to other known interacting proteins, including TSG101 (Tumor susceptibility gene 101) and annexin A11. The X-ray crystal structure of the F122A mutant revealed that removal of the bulky F122 side chain not only created an additional open space in Pocket 2 but also abolished inter-helix interactions with W95 and V98 (present in α4) and that α5 inclined away from α4 to expand Pocket 2, suggesting acquirement of more appropriate positioning of the interacting residues to accept Alix.

**Conclusions:**

We found that the inability of the two-residue shorter ALG-2 isoform to bind Alix is not due to the absence of bulky side chain of F122 but due to deformation of a main-chain wall facing pockets 1 and 2. Moreover, a residue at the position of F122 contributes to target specificity and a smaller side chain is preferable for Alix binding but not favored to bind annexin A11.

## Background

ALG-2 (apoptosis-linked gene 2) is a 22-kDa protein of 191 amino acid residues containing five serially repetitive EF-hand-type helix-loop-helix Ca^2+^-binding motifs (EF1 to EF5) and it belongs to the penta-EF-hand (PEF) family, including the calpain small subunit, sorcin, grancalcin and peflin in mammals [1]. ALG-2 is the most conserved protein among the PEF family and its homologues are widely found in eukaryotes. Despite the original report of a pro-apoptotic function of ALG-2 in T cell hybridomas [2], ALG-2-deficient mice develop normally with no obvious abnormalities in the immune system [3]. Nonetheless, potential physiological roles of ALG-2 in control of ER-stress-induced apoptosis, cancer and cell division have been reported [4-6]. Alix (also named AIP1) was the first protein identified as an ALG-2-interacting protein [7,8]. This cytoplasmic 95-kDa protein is now recognized as an auxiliary factor of the ESCRT (endosomal sorting complex required for transport) system, which is involved in endosomal sorting, retrovirus budding and cytokinesis [9-11]. In addition to roles in the ESCRT system, Alix functions in actin-cytoskeleton assembly, cell adhesion, signal transduction and apoptosis [12-15].

X-ray crystal structures of various PEF proteins including ALG-2 have common features: the presence of eight α-helices and dimer formation via paired EF5s that are positioned in anti-parallel orientation [16-20]. Previously, we solved the structures of Ca^2+^-free and -bound forms of N-terminally truncated human ALG-2 (des3-20ALG-2) and a Zn^2+^-bound form of full-length ALG-2 as well as the structure of the complex between des3-23ALG-2 and the peptide corresponding to Alix799-814 in the Zn^2+^-bound form. Although the four-EF-hand-region (EF1-EF4) of ALG-2 has a general structural resemblance to calmodulin, ALG-2 exhibits only a very small Ca^2+^-dependent conformational change. Binding of Ca^2+ ^(or Zn^2+^) to EF3 enables the side chain of R125, present in the loop connecting EF3 and EF4, to move enough to make a primary hydrophobic pocket (Pocket 1) accessible to the critical PPYP motif found in Alix. This Ca^2+^/EF3-driven arginine switch mechanism explains how ALG-2 is activated by Ca^2+ ^to bind to its target proteins [21,22]. The C-terminal half of the Alix peptide is also held in the second hydrophobic pocket (Pocket 2). On the other hand, in the case of calmodulin, each pair of EF1-EF2 (N-lobe) and EF3-EF4 (C-lobe) changes its conformation from "closed" to "open" state upon Ca^2+ ^binding and exhibits a further gross change in relative stereotypic position by bending of the central helix connecting EF2 and EF3 in such a way that the two lobes grab the targeting peptide [23].

An isoform of ALG-2 was first reported in the mouse [24]. The isolated cDNA clone designated ALG-2,1 was shorter in six nucleotides corresponding to the two amino acids Gly^121^Phe^122 ^in comparison with the full-length cDNA clone ALG-2,5. Both transcripts were present in mouse tissues at an approximate ratio of 2:1 (ALG-2,5 *vs *ALG-2,1). The same type of isoform lacking Gly^121 ^Phe^122 ^(designated ALG-2^ΔGF122 ^in this article; non-deleted ALG-2 being regarded as wild type for convenience) is also registered in human DNA databases, such as GenBank under accession no. BC110291.1. Interaction of ALG-2^ΔGF122 ^with Alix is significantly reduced or not detected in yeast two-hybrid or *in vitro *binding assays [24-27]. Although F122 interacts with the ALG-2-binding site (ABS) peptide of Alix in Pocket 2 in the crystal structure [21], the molecular basis for the defect of ALG-2^ΔGF122 ^in binding to Alix has remained to be elucidated. In the present study, we crystallized des3-23ALG-2^ΔGF122 ^and compared its X-ray crystal structure with that of the Ca^2+^-bound form of des3-23ALG-2. We found that deletion of the two residues causes shortening of an α-helix (α5) and leads to a change in the configuration of the R125 side chain. Surprisingly, however, the F122A mutant (ALG-2^F122A^, Phe substituted with Ala) exhibited an unexpected hyperactivity in Alix-binding. We also investigated effects of this mutation on the crystal structure, and we discuss the structural roles of F122 in this article.

## Results

### Structures of ALG-2^ΔGF122 ^and ALG-2^F122A^

For determination of the 3D structures of ALG-2^ΔGF122 ^and ALG-2^F122A^, we prepared recombinant proteins with deletion in the N-terminal Gly/Pro-rich region (des3-23ALG-2^ΔGF122 ^and des3-20ALG-2^F122A^, respectively). Crystal structures were solved by the molecular replacement method using the previously solved structures of ALG-2 (PDB IDs 1HQV and 2ZN8) as a search model. Data collection, processing, and refinement statistics are summarized in Table 1. The structures of des3-23ALG-2^ΔGF122 ^in the Ca^2+^-bound form (PDB ID, 3AAJ) and des3-20ALG-2^F122A ^in the Zn^2+^-bound form (PDB ID, 3AAK) were solved at resolutions of 2.4 Å and 2.7 Å, respectively. Although the obtained data of 3AAK were processed to 2.5 Å, the refinement gave poor R_work _and R_free _values (near or greater than 30). Thus, we limited the resolution to 2.7 Å (R_work_/R_free_, 25.2/29.8). An asymmetric unit of the crystal of des3-23ALG-2^ΔGF122 ^in the Ca^2+^-bound form contained two ALG-2 molecules (A and B) as a dimer (Additional file 1, Figure S1). The root-mean-square deviation (rmsd) value of the structures aligned between two molecules was calculated to be 0.73 Å for C^α ^atoms from residues L28 to V189. The structure of molecule A was used for further analysis. Crystals of the Ca^2+^-free and Ca^2+^-bound forms of des3-20ALG-2^F122A ^suitable for X-ray diffraction were not obtained.

**Table 1:** Data collection and refinement statistics

	des3-23ALG-2^ΔGF122^/Ca^2+^-bound	des3-20ALG-2^F122A^/Zn^2+^-bound
PDB code	3AAJ	3AAK
**Data collection**		
Beamline	PF BL-5A	PF AR NW-12
Wavelength (Å)^a^	1	1
Space group	*P*2_1_	*C*222_1_
a/b/c (Å)^a^	42.6/78.9/60.3	71.3/170.2/47.0
α/β/γ (°)^a^	90.0/104.6/90.0	90.0/90.0/90.0
Resolution (Å)^a^	50.0-2.40(2.49-2.40)	50.0-2.50(2.59-2.50)
Measured reflections	55354	70399
Unique reflections	15185	10302
Completeness (%)^a^	97.9(95.1)	97.3(88.4)
R_merge _(%)^a^	5.3(18.8)	4.3(12.0)
I/σ^a^	15.5(5.8)	28.8(14.3)
**Refinement**		
Resolution (Å)	50.0-2.40	50.0-2.70
R_work_/R_free _(%)	19.6/26.6	25.2/29.8
Rmsd bond length (Å)	0.019	0.006
Rmsd bond angle (°)	1.68	0.86
Average B-factor (Å^2^)	36.8	56.3
No. ALG-2 molecule	2	1

^a ^Values in parentheses are for highest-resolution shell.

**Figure 1:**
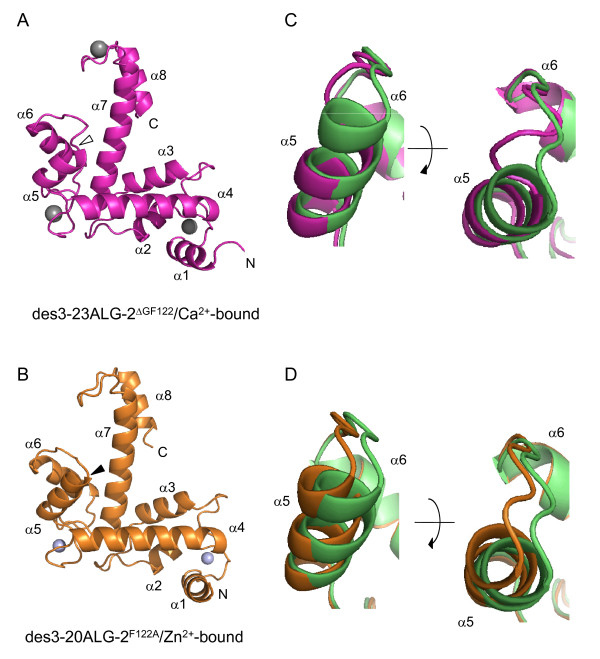
**Structures of ALG-2^ΔGF122 ^and ALG-2^F122A^**. Structures of (A) Ca^2+^-bound des3-23ALG-2^ΔGF122 ^(PDB ID 3AAJ) and (B) Zn^2+^-bound des3-20ALG-2^F122A ^(PDB ID 3AAK) are shown in magenta and orange, respectively, in ribbon representations, and EF-hand-coordinated calcium and zinc ions are shown in gray and light cyan spheres. The deleted site (Gly^121^Phe^122^) and substituted site (F122A) are marked by an open arrowhead and closed arrowhead in (A) and (B), respectively. (C, D) The ALG-2 structures are compared with the previously resolved structures of wild-type ALG-2 of Ca^2+^-bound form (PDB ID 2ZN9) and Zn^2+^-bound form (PDB ID 2ZN8) by aligning at α-helix 4 (α4), and close-up views of segments from α5 to α6 are shown in ribbon representations. (C) The structures of Ca^2+^-bound des3-20ALG-2 and des3-23ALG-2^ΔGF122 ^are shown in green and magenta, respectively. The view in the left panel is rotated approximately 90° to view from the top as shown in the right panel. (D) The structures of Zn^2+^-bound ALG-2 (green) and des3-20ALG-2^F122A ^(orange) are presented similarly to (C).

**Figure 2:**
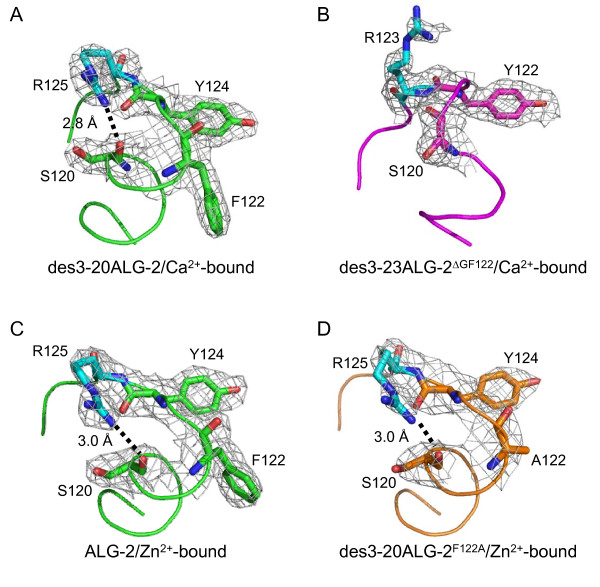
**Differences in the side chain configurations of R125 and R123**. Close-up views of a segment from L115 to D128 (D126 in des3-23ALG-2^ΔGF122^) in each ALG-2 structure are shown in ribbon representations and in stick models superimposed with electron densities of residues that are involved in the Ca^2+^/EF3-driven arginine switch mechanism. The structures of (A, C) wild-type ALG-2, (B) des3-23ALG-2^ΔGF122 ^and (D) des3-20ALG-2^F122A ^are colored green, magenta and orange, respectively. R125, a critical residue for the switch mechanism and interaction with Alix, and its corresponding residue in des3-23ALG-2^ΔGF122 ^(R123) are colored cyan. The hydrogen bond between the guanidino nitrogen atom of R125 and carbonyl oxgen atom of S120 is shown by a dotted line and the distance is indicated in (A), (C) and (D).

The basic architectures of the PEF domain containing eight α-helices (α1-α8), five EF-hand-like helix-loop-helix motifs (EF1-EF5), and pairing at EF5 as a dimer were maintained in the solved crystal structures (Figure 1 and Additional file 1, Figure S1), and the overall structures were very similar when compared with those of wild-type ALG-2 in the Ca^2+^-bound form of des3-20ALG-2 (PDB ID 2ZN9) and the Zn^2+^-bound form of full-length ALG-2 (PDB ID 2ZN8), respectively (rmsd values: des3-23ALG-2^ΔGF122^/Ca^2+ ^*vs *des3-20ALG/Ca^2+^, 1.23 Å for C^α ^atoms from residues 24-189 of ALG-2; des3-20ALG^F122A^/Zn^2+ ^*vs *ALG-2/Zn^2+^, 0.62 Å for C^α ^atoms from residues 26-191; des3-20ALG/Ca^2+ ^vs ALG-2/Zn^2+^, 0.76 Å for C^α ^atoms from residues 26-189). In des3-23ALG-2^ΔGF122^, however, the deletion of Gly^121^Phe^122 ^caused loss of the third turn in α5 that corresponds to the exiting helix of EF3 (Figure 1C, magenta). The loop connecting α5 of EF3 and α6 of EF4 started earlier, but it returned to a similar spatial position in the middle of the loop around Y122, corresponding to Y124 in wild-type ALG-2 (Figure 2A and 2B). This spatial position of Y122 was supported partly by hydrogen bonding between the nitrogen atom of Y122 (N^Y122^) and the peptide carbonyl oxygen atom of L119 (O^L119^) and partly by hydrophobic interactions between C^β ^of Y122 and C^δ1 ^of L124 as in the case of Y124 of wild-type ALG-2 (denoted CB and CD1, respectively, in the Latin alphabet in Table 2). While hydrogen bonding between the hydroxyl oxygen atom (O^η^) of Y122 (OH^Y122^) and the side chain amide nitrogen atom (N^ε2^) of Q157 (NE2^Q157^) in α7 was newly formed, hydrophobic interactions between side chains of Y122 and L156 were reduced in the ΔGF122 isoform. In the crystal structure of des3-20ALG-2^F122A ^in the Zn^2+^-bound form, the axis of α5 inclined slightly outward from α4 and α7 (see Discussion section) (Figure 1D, orange).

**Table 2:** Intramolecular interactions of ALG-2 in Ca^2+^/EF3-driven arginine switching loop

		WT		ΔGF122
		
	interacting atom	**MF **^**a**^	**Ca **^**b**^	**Ca **^**c**^
S120				
Hydrogen		Distance (Å)		
		
N	O^K116^	2.8	3.2	3.1
O	N^G123/121^	2.8	2.8	ND^d^
	NH2^R125/123^	ND	2.8	ND
OG	O^L116^	2.6	ND	ND
Y124/122				
hydrogen				
N	O^L119^	3.1	3.0	3.0
OH	NE2^Q159/157^	ND	ND	2.8
hydrophobic				
CB	C^L119^	ND	3.9	ND
	CD1^L126/124^	3.9	3.7	3.9
	CG^L126/124^	ND	ND	3.7
CD1	CB^F122/-^	3.7	3.6	-^e^
	CD1^L158/156^	ND	ND	4.0
CD2	CD2^L158/156^	3.9	ND	ND
CE1	CB^F122/-^	3.8	3.8	-
	CD1^F122/-^	3.9	ND	-
	CD2^L158/156^	3.9	ND	ND
CE2	CG^L158/156^	3.5	ND	ND
	CD2^L158/156^	3.7	3.8	ND
CZ	CD1^L158/156^	ND	4.0	ND

^a ^Metal-free form of des3-20ALG-2

^b ^Ca^2+^-bound form of des3-20ALG-2

^c ^Ca^2+^-bound form of des3-23ALG-2^ΔGF122^

^d ^Not detected in the range of 2.6-3.2 Å for hydrogen bonding and 3.2-4.0 Å for hydrophobic interaction

^e ^Not present in des3-23 ALG-2^ΔGF122^

### Configuration of R125 side chain

In the previously proposed Ca^2+^/EF3-driven arginine switch mechanism [21,22], binding of Ca^2+ ^to EF3 enables formation of a hydrogen bond between one of the guanidino nitrogen atoms (N^η2^) of R125 (NH2^R125^) and the peptide carbonyl oxygen atom of S120 (O^S120^), resulting in change in the configuration of the R125 side chain (Table 2, Figure 2, A and 2B; Additional file 1, Figure S2A). Binding of Zn^2+ ^caused the same effect (Figure 2C) [21]. In the structure of des3-23ALG-2^ΔGF122 ^in the Ca^2+^-bound form, however, NH2^R123 ^(corresponding to NH2^R125 ^in wild-type ALG-2) did not form such a hydrogen bond (Figure 2B). The side chain extended outwardly from the loop as in the case of the structure of the metal-free form, but the spatial positions of the side chains between R123 and Y122 were closer in the equivalent residues in the metal-free ALG-2 (R125 and Y124) (Additional file 1, Figure S2A). The lower electron densities corresponding to guanidino group atoms of R123 suggest flexibility of this side chain by solvent exposure. On the other hand, NH2^R125 ^in the structure of Zn^2+^-bound des3-20ALG-2^F122A ^formed a hydrogen bond with O^S120^, and the side chain of R125 showed a configuration similar to that of wild-type ALG-2 (Figure 2, C and 2D).

### Metal coordination

Three calcium ions were found in EF1, EF3 and EF5 of des3-23ALG-2^ΔGF122 ^at the canonical EF-hand coordination positions (x, y, z, -y, -x and -z), though water molecules at the -x positions in EF1 and EF3 were absent and no amino acid residue coordinated at the -z position in EF5 (Additional file 1, Figure S3; Additional file 2, Table S1). In addition to two zinc ions found in EF1 and EF3 at the canonical coordination positions (Additional file 2, Table S2), one zinc ion that was coordinated at a non-canonical position was found in EF5 in the structure of Zn^2+^-bound des3-20ALG-2^F122A ^(Additional file 1, Figure S4; Additional file 2, Table S3). In this non-canonical EF-hand coordination, zinc ion was bonded to OD1^D171 ^and OD2^D173 ^in place of OD1^D169 ^and O^W175^, respectively, at the x and -y positions. Interestingly, the Ca^2+^-bound form and the Zn^2+^-bound form displayed an opposite relationship regarding the presence of water molecules at -x positions in wild-type ALG-2 between EF1 and EF3 (water molecules at -x: EF1/Ca^2+^, present *vs *EF1/Zn^2+^, absent; EF3/Ca^2+^, absent *vs *EF3/Zn^2+^, present) (Additional file 2, Table S1; Additional file 2, Table S2). In the Zn^2+^-bound form of ALG-2^F122A^, a water molecule was found at this position in EF1. The difference in ion radius between calcium and zinc (0.99 Å and 0.74 Å, respectively) may influence the availability of coordinated water molecules at the -x position.

### Comparison of surface structures

**Figure 3:**
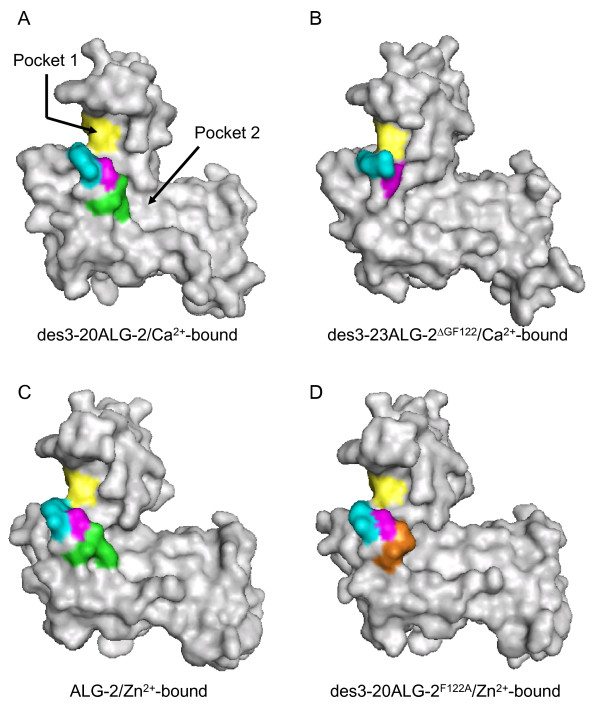
**Loss of a wall surrounding hydrophobic pockets in ALG-2^ΔGF122^**. Surface structures of (A) Ca^2+^-bound des3-20ALG-2, (B) Ca^2+^-bound des3-23ALG-2^ΔGF122^, (C) Zn^2+^-bound ALG-2, and (D) Zn^2+^-bound des3-20ALG-2^F122A ^are presented in gray except for indicated residues of Gly^121^Phe^122 ^(green), G123 (or G121 in des3-23ALG-2^ΔGF122^) (magenta), A122 in the F122A mutant (orange), R125 (or R123 in des3-23ALG-2^ΔGF122^) (cyan), Y180 (or Y178 of des3-23ALG-2^ΔGF122^) from a dimerized counterpart molecule (yellow).

As shown in Figure 3, the deletion of two residues caused a noticeable change in the surface structure surrounding hydrophobic pockets (designated Pocket 1 and Pocket 2), which were shown to accommodate the Alix ABS peptide in our previous study [21]. The bottom of Pocket 1 was supported by Y180 derived from a dimerizing counterpart molecule of ALG-2 (Figure 3, yellow). A wall formed by Gly^121^Phe^122 ^in des3-20ALG-2/Ca^2+^-bound (Figure 3A, green) disappeared in des3-23ALG-2^ΔGF122^/Ca^2+^-bound, and a surface structure represented by G123 was also changed (Figure 3B, magenta) The continuous wall formed by 121-GFG-123 was maintained in Zn^2+^-bound des3-20ALG-2^F122A ^(Figure 3D, orange: Gly^121^Ala^122^; magenta, G123). The R125 side chain (Figure 3, cyan) was oriented away from Pocket 1 in the metal-bound ALG-2 proteins (Figure 3, A, C and 3D). On the other hand, the side chain directed toward Pocket 1 in Ca^2+^-bound des3-23ALG-2^ΔGF122 ^(Figure 3B), but it did not fully block the entry path of Pocket 1 as in the case of metal-free ALG-2 (Additional file 1, Figure S2B).

### Effects of amino acid substitutions of F122 on binding capacities and specificities

To investigate whether the side chain of F122 influences association between ALG-2 and Alix, we performed real-time surface plasmon resonance (SPR) interaction analyses using purified recombinant full-length ALG-2 and F122-substituted mutants as well as ALG-2^ΔGF122 ^as analytes and an Alix ABS peptide as a ligand. The resonance signals at 10 s before the dissociation phase were compared with that of wild-type (WT) ALG-2 and expressed as relative binding capacities (Additional file 1, Figure S5). Higher binding capacities were observed for the ALG-2 mutants of F122A (240%) and F122G (140%), whereas lower binding capacities were observed for ALG-2^ΔGF122 ^(18%) and the mutants of F122W (36%) and F122S (89%).

**Figure 4:**
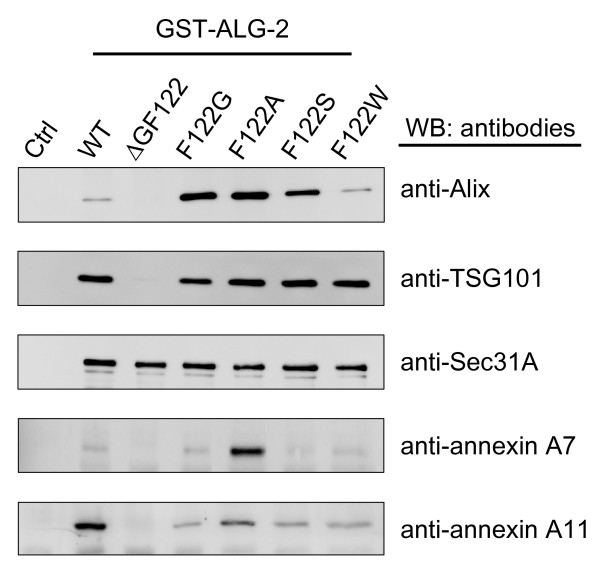
**Effects of amino acid substitutions of F122 on binding specificities**. ALG-2-interacting proteins in HEK293 cells were pulled down with GST-fused ALG-2 of wild type (WT), ALG-2^ΔGF122 ^(ΔGF122), and F122-substituted mutants. Unfused GST was used as a negative control (Ctrl). Proteins bound to glutathione Sepharose beads were subjected to Western blotting with antibodies against Alix, TSG101, Sec31A, annexin A7, and annexin A11.

Next, we investigated whether substitutions of F122 equally influence binding to endogenous ALG-2-interacting proteins by pulldown assays using glutathione-*S*-transferase (GST)-fused ALG-2 proteins and unfused GST as a negative control (Ctrl) (Figure 4). Pulldown products were analyzed by Western blotting with specific antibodies against Alix, TSG101, Sec31A, annexin A7 and annexin A11. GST-ALG-2^ΔGF122 ^gave essentially no specific immunoreactive signals except for Sec31A. For Alix interaction, the mutants of small side chain substitutions (F122G, F122A and F122S) gave stronger signals than those of WT and F122W mutant. Compared to the results obtained by SPR analyses (Additional file 1, Figure S5), GST-pulldown assays gave much more enhanced signals for mutants than for WT. Even F122W mutant gave a capacity similar to that of WT. Capacities of ALG-2 binding to TSG101 and Sec31A were not different from WT in all F122 mutants. Compared to WT, only F122A mutant gave significantly stronger signals for annexin A7, whereas signals for annexin A11 were decreased in all mutants.

### Augmentation of staurosporine-induced cell death by expression of ALG-2^F122A^

**Figure 5:**
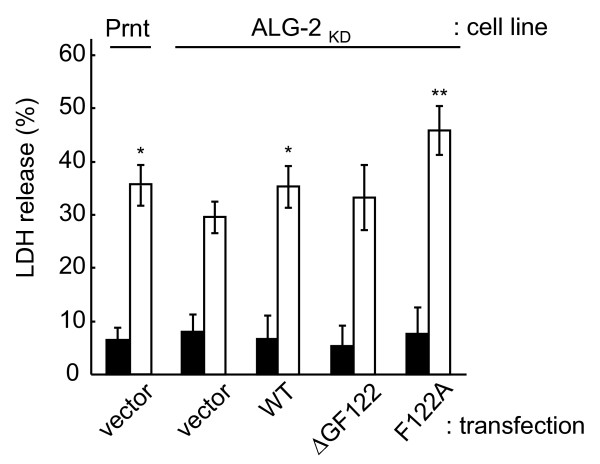
**Augmentation of staurosporine-induced cell death by ALG-2^F122A^**. Parental (Prnt) HeLa cells and ALG-2-knockdown (ALG-2_KD_) HeLa cells were transfected with a vector (pcDNA3) as controls. ALG-2_KD _cells were transfected with plasmids that express wild-type ALG-2, ALG-2^ΔGF122 ^or ALG-2^F122A ^and cultured for 24 h. The transfected cells were treated with 1 μM staurosporine for 24 h. Cell death was monitored by measuring the amounts of lactate dehydrogenase (LDH) released into the culture medium as described in Materials and Methods. Total amounts of LDH released into the medium and retained in cells were expressed as 100%. Duplicate assays of five independent repetitive experiments were performed and data are presented as means +/- SD (n = 5). Closed column, not treated with staurosporine; open column, treated with staurosporine. Statistically significant differences among staurosporine-treated cells were evaluated by Student's t-test by comparing the measured values of samples with those of control ALG-2_KD _cells that were transfected with a control vector (*, P < 0.05; **, P < 0.01)

Staurosporine, a microbial alkaloid, acts as a non-selective protein kinase inhibitor with high potency by binding to ATP-binding pockets of kinases [28], and it induces cell death via caspase-dependent and -independent apoptotic pathways [29]. Previously, Vito *et al. *reported that ALG-2 and Alix (named AIP1 in the article) modulate staurosporine-induced cell death [8]. To investigate whether the enhanced Alix-binding capacity by F122A mutant exerts augmentation of cell death, we employed previously established ALG-2-knockdown (ALG-2_KD_) HeLa cells whose endogenous ALG-2 level was reduced by the RNA interference (RNAi) method [26]. After transfection with RNAi-resistant expression plasmids of either wild-type or mutant ALG-2 proteins or with a vector as a control, cells were treated with staurosporine for 24 h. The degree of cell death was estimated by measuring the amounts of lactate dehydrogenase (LDH) released into the culture medium. As shown in Figure 5, cells not treated with staurosporine released small amounts of LDH under the conditions used (closed columns, 6-8% of total LDH activities). On the other hand, dramatic increases were observed for the release of LDH from staurosporine-treated cells in all cases tested (open columns, 29-46%), but vector-transfected ALG-2_KD _HeLa cells (29%) showed lower values than vector-transfected parental HeLa cells (36%). The amounts of released LDH were increased slightly by exogenous expressions of wild-type (WT) ALG-2 (36%) and ALG-2^ΔGF122 ^(33%) in ALG-2_KD _HeLa cells. The augmentation was statistically significant (P < 0.01) when ALG-2^F122A ^was exogenously expressed (46%) and compared with the vector-transfected ALG-2_KD _HeLa cells (29%).

## Discussion

A variant of ALG-2 cDNA lacking six nucleotides is found in human DNA databases. Since Gly^121^Phe^122 ^is immediately followed by Gly in the amino acid sequence, the deletion of Phe^122^Gly^123 ^is assigned in the results of BLAST searches using the blastp or tblastn program. In the nucleotide sequence of cDNAs, however, the deletion occurs in GGTTTC (Gly^121^Phe^122^) but not in TTCGGC (Phe^122^Gly^123^) in the sequence of TCAGGTTTCGGCTAC (corresponding to 120-SGFGY-124). In the current human EST database, the ratio of retrieved human ALG-2 cDNAs containing nucleotides corresponding to Gly^121^Phe^122 ^and those lacking the corresponding hexanucleotides is approximately 3:1. In this article, we designate the major ALG-2 isoform as wild type (WT) to discriminate it from the shorter and minor isoform. Analysis of the human genome database revealed that the *ALG-2 *gene (symbol, *PDCD6*; location, 5 pter-p15.2) has six exons and that the boundary sequence of Exon 4 and Intron 4 contains an alternative splicing donor site (TCAG**GTTTCG**gtaactcactcactc: lower case, intron sequence; boldfaced sequence, missing by alternative splicing). The alternatively spliced ALG-2 isoform, designated ALG-2^ΔGF122^, is defective in Alix-binding [24-26]. These two residues (Gly^121^Phe^122^) comprise the last turn in α5 in the crystal structures [18,21]. However, the structural basis for the inability of ALG-2^ΔGF122 ^to bind Alix has remained to be established. In the present study, we investigated the role of Gly^121^Phe^122 ^by solving the X-ray crystal structure of the shorter isoform and by mutational analyses of F122.

**Figure 6:**
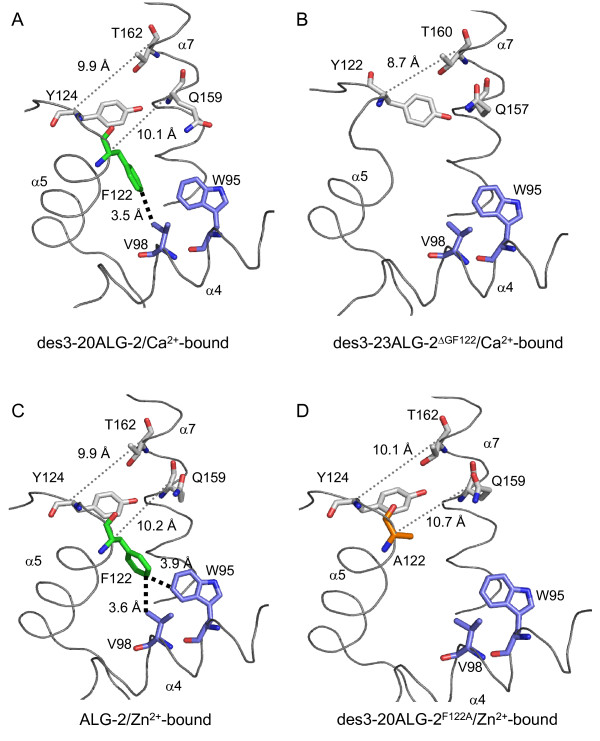
**Close-up views of structural interrelations among α4, α5 and α7**. Residues influencing space size in the hydrophobic pockets are shown in stick models and described in Discussion. Thick dotted lines represent hydrophobic interactions. Thin dotted lines indicate C^α^-distances that show differences in the pockets between wild-type and mutant ALG-2 structures. In the stick model representation, carbons atoms are colored in blue for W95 and V98, green for F122, orange for A122 and gray for Y124, Q159 and T162.

Comparison of the crystal structures of des3-20ALG-2 and des3-23ALG-2^ΔGF122 ^in the Ca^2+^-bound forms revealed a significant difference in spatial positions of residues adjacent to Gly^121^Phe^122 ^(Figures 1, 2, 3). Since S120 is located within α5 in ALG-2 but is placed at the end of the truncated helix in ALG-2^ΔGF122 ^(Figure 1), an α-helix-supporting hydrogen bond between O^S120 ^and N^G121 ^(G123 in wild type) is disrupted (Table 2). This causes a change in the spatial orientation of O^S120^, and the hydrogen bond with NH2^R125 ^in wild-type ALG-2 is lost in NH2^R123 ^in ALG-2^ΔGF122^. Then, the side chain of R123 is placed to partially block Pocket 1, which is a primary acceptor site for the Alix ABS peptide (Figures 2 and 3) and resembles the topology of R125 in the Ca^2+^-unbound (metal-free) form of wild-type ALG-2 (Additional file 1, Figure S2). F122, present in α5 (exiting α-helix of EF3), interacts with residues W95 and V98 in α4 (entering α-helix of EF3) and stabilizes the EF-hand motif in metal-free des3-20ALG-2 (Table 3 and Additional file 1, Figure S2C,). This inter-helix interaction is maintained partly in the Ca^2+^-bound form (F122-V98 interaction) and disrupted in Ca^2+^-bound ALG-2^ΔGF122 ^due to the absence of F122 (Figure 6, A and 6B, Table 3). The distance between the C^α ^atoms of Y122 and T160 (facing Pocket 1) is shorter in the crystal structure of des3-23ALG-2^ΔGF122 ^(Figure 6B, 8.7 Å) than that between the corresponding Y124 and T162 in the Ca^2+^-bound (Figure 6A, 9.9 Å) and metal-free structure of des3-20ALG-2 (Additional file 1, Figure S2C, 9.0 Å), indicating a narrower Pocket 1 in the isoform. Moreover, the main chain of G123 in wild-type ALG-2 (Figure 3, magenta) is placed to face Pocket 1, and the main chain carbonyl carbon (C') interacts hydrophobically with the Alix ABS peptide [21], but this interaction is no longer possible because a continuous wall formed by Gly^121^Phe^122 ^(facing Pocket 2) and G123 (facing Pocket 1) is destroyed in ALG-2^ΔGF122 ^(Figure 3).

**Table 3:** Loss of intramolecular interactions in F122A mutant

		**WT**			**F122A**
		
	**interacting atom**	**MF**^**a**^	**Ca**^**b**^	**Zn**^**c**^	**Zn**^**d**^
	
		**Distance (Å)**			
		
F122					A122
hydrogen					
N	O^A118^	ND	2.9	3.2	ND^e^
	O^L119^	3.0	3.1	ND	3.2
hydrophobic					
CB	CD1^Y124^	3.7	3.6	3.9	ND
	CE1^Y124^	3.8	3.8	4.0	ND
CD1	C^A118^	ND	ND	4.0	-^f^
	CA^L119^	3.9	ND	4.0	-
	CD2^L119^	3.9	3.7	4.0	-
	CE1^Y124^	3.9	ND	ND	-
CE1	CH2^W95^	3.9	ND	ND	-
	CZ3^W95^	3.9	ND	ND	-
CE2	CG1^V98^	ND	4.0	ND	-
CZ	CH2^W95^	3.9	ND	ND	-
	CZ3^W95^	3.5	ND	3.9	-
	CG1^V98^	3.8	3.5	3.6	-

^a ^Metal-free form of des3-20ALG-2

^b ^Ca^2+^-bound form of des3-20ALG-2

^c ^Zn^2+^-bound form of ALG-2

^d ^Zn^2+^-bound form of des3-20ALG-2^F122A^

^e ^Not detected in the range of 2.6-3.2 Å for hydrogen bonding and 3.2-4.0 Å for hydrophobic interaction

^f ^Not present in des3-20ALG-2^F122A^

Our previous X-ray crystal structure analyses of the complex between ALG-2 and the Alix ABS peptide (1-QG**PPYP**TYPG**YP**GYSQ-16, core motif **boldfaced**) revealed that an aromatic moiety of F122, constituting Pocket 2, interacts hydrophobically with Y11 of the Alix ABS peptide [21]. Surprisingly, however, substitution of F122 with Ala or Gly did not abolish the binding but instead increased the binding capacities as shown by SPR analyses, and substitution with Trp caused a decrease in the binding capacity (Additional file 1, Figure S5). Similar effects were observed by GST-pulldown assays of endogenous Alix (Figure 4). Thus, it is thought that the inability of ALG-2^ΔGF122 ^to bind Alix is not due to the absence of a residue with a large hydrophobic side chain at residue No. 122 but is due to the unfavorable topology of the loop connecting α5 and α6 caused by deletion of the two residues. This idea may also explain the finding by Subramanian *et al. *that a single amino acid deletion of F122 caused a marked decrease in Alix-binding capacity [30]. On the other hand, the presence of an aromatic bulky side chain at this position (in Pocket 2) may be rather detrimental for binding to Alix due to steric hindrance. Interestingly, substitution of either M71 or Q159 (both present in Pocket 2 and interacting with the Alix ABS peptide in the complex crystal structure) with Ala resulted in higher Alix-binding capacities [21]. Thus, more open space or a deeper trough in Pocket 2 seems to favor interactions with Alix. The F122S mutant showed lower binding capacity (89%) than that of wild-type ALG-2 by SPR analyses (Additional file 1, Figure S5), but the GST-ALG-2^F122S ^mutant exhibited a higher capacity than that of the wild type in the GST-pulldown assay. This discrepancy may arise from the differences in the ligands to be assayed (SPR: ABS oligopeptide corresponding to residue No. 799-814 of Alix; GST-pulldown: endogenous Alix protein of 868 residues) because residues 815-842 also contribute to interactions with ALG-2 to some extent [31].

EF-hand proteins have similar helix-loop-helix structures, but conformational states with respect to angles and distances between the two helices and their changes in response to Ca^2+ ^binding are very diverse [32]. Unlike calmodulin, ALG-2 does not exhibit a significant change from the closed conformational state in the absence of Ca^2+ ^to the open conformational state in the presence Ca^2+ ^[21]. Nevertheless, binding of Ca^2+ ^or Zn^2+ ^to EF3 causes a small shift of α5 and leads to change in configuration of the R125 side chain [21]. Substitution of F122 with Ala disrupts the inter-helix interaction between α4 and α5 (Figure 6, C and 6D). Comparison of the estimated angles formed by α4 (entering helix) and α5 (exiting helix) in EF3 between the structures of wild-type ALG-2 (θ = 54.5°) and des3-20ALG-2^F122A ^(θ = 62.6°) in the Zn^2+^-bound forms (Table 4) indicates inclination of α5 by 8.1° away from α4, resulting in a shift in the position of the C^α ^atom of F122A for 2.0 Å (Figure 1D). This more open conformation of the EF-hand motif in F122A mutant maintains distances or causes a small increase in distances between the C^α ^atoms of Y124 and T162 (facing Pocket 1) (WT, 9.9 Å; F122A, 10.1 Å) and between the C^α ^atoms of F122/A122 and Q159 (facing Pocket 2) (WT, 10.2 Å; F122A, 10.7 Å). On the other hand, regardless of the disruption of inter-helix interaction, ALG-2^ΔGF122 ^gave only a small increase in the angle (WT, θ = 57.6°; ΔGF122, θ = 58.6°) in the Ca^2+^-bound form structure (Table 4), and the distance between the C^α ^atoms of Y124 and T160 was significantly decreased in ALG-2^ΔGF122 ^(Figure 6, WT, 9.9 Å; ΔGF122, 8.7 Å). Consequently, the change in the relative spatial positions of α5 and the following loop may allow the side chains of A122, Y124 and adjacent R125 to place at more appropriate positions in the hydrophobic pockets of ALG-2^F122A ^to interact with the Alix ABS peptide. To corroborate the above hypothetical mechanism of enhanced binding of ALG-2^F122A ^to Alix, we attempted co-crystallization of des3-20ALG-2^F122A ^with the Alix ABS peptide but could not obtain good crystals. The nature of N-terminal residues of ALG-2 influences qualities of crystals. In the present study, we used des3-20ALG-2^F122A ^because X-ray diffraction resolutions were better in des3-20ALG-2 (2.4 Å) than in des3-23ALG-2 (3.1 Å) in the Ca^2+^-bound forms in our previous study [33]. It would be worthwhile, however, to test des3-23ALG-2^F122A ^for crystal preparations of the complex.

**Table 4:** Angles and distances calculated for ALG-2 EF3. ALG-2 EF3 has α4 (entering helix, residue No. 93-102) and α5 (exiting helix, residue No. 112-122), but a segment of α5 (residue No. 112-120, common to ALG-2^ΔGF122^) was used.

		Angle (°)			Distance (Å)		
		
		θ	φ	ω	inner ends	midpoint	outer ends
WT	MF^a^	49.5	104	137	10.6	12.2	14.7
	Ca^b^	57.6	96.9	131	10.7	12.7	15.6
	Zn^c^	54.5	98.0	130	10.7	12.6	15.4
ΔGF122	Ca^d^	58.6	88.8	134	10.7	13.1	16.3
F122A	Zn^e^	62.6	97.6	128	11.0	13.2	16.4

^a ^Metal-free form of des3-20ALG-2

^b ^Ca^2+^-bound form of des3-20ALG-2

^c ^Zn^2+^-bound form of ALG-2

^d ^Ca^2+^-bound form of des3-23ALG-2^ΔGF122^

^e ^Zn^2+^-bound form of des3-20ALG-2^F122A^

Angles and distances were estimated by the Vector Geometry Mapping (VGM) software (Ref. 50)

ALG-2 interacts with various proteins with Pro-rich regions [34]. Previously, we classified ALG-2-interacting proteins into two groups (Alix type and non-Alix type) based on ability of binding to ALG-2^ΔGF122 ^[27]. Alix-type proteins such as TSG101, annexin A7 and annexin A11 contain a consensus sequence of PPYPX_3-5_YP (where X is uncharged residues and PPYP is substitutable with PXYP or XPYP) similar to the core motif of Alix [21]. On the other hand, ALG-2^ΔGF122 ^retains binding ability to non-Alix-type proteins such as Sec31A and phospholipid scramblase 3 (PLSCR3) that possess a common sequence of PXPGF (where X is P or A) but whose indispensability remains to be experimentally verified [26,27]. The mutation of F122 had different effects on capacities of binding to endogenous proteins among Alix-type proteins, even to two paralogous annexin family proteins (Figure 4). Although ALG-2-binding sites still remain to be identified, annexin A7 and annexin A11 have 4-PGYPPTGYPP-13 and 4-PGYPPPPGGYPP-15, respectively. A clear difference between the two sequences is the number of residues present between two YPs (annexin A7, 3 *vs *annexin A11, 5). Since F122 constitutes Pocket 2 and interacts with the aromatic side chain of the corresponding second YP in the Alix ABS peptide, mutation of F122 seems favored or disfavored for interaction depending on spatial positions of the concerned Tyr residues in the target proteins. Recently, mucolipin-1 has been shown to Ca^2+^-dependently interact with ALG-2 but not with ALG-2^ΔGF122 ^[35]. The predicted binding site (37-EEDLRRRLKYFF-49) does not contain any YP or PXPGF motif, but charged residues as well as hydrophobic residues were shown to be important for interactions. Thus, mucolipin-1 may be recognized by a surface different from that for binding to Pro-rich target proteins. Ask1 and Raf-1, which are also known to interact with ALG-2 [36,37], do not possess conspicuous Pro-rich regions either, and the ALG-2-binding site has not been reported yet. It would be interesting to see whether F122A and other amino acid-substituted mutants of Pocket 1 and Pocket 2 present a different binding profile between Pro-rich type and non-Pro-rich type ALG-2-interacting proteins.

The biological significance of the occurrence of ALG-2^ΔGF122 ^is not known. ALG-2 forms a dimer and each molecule of the dimer has capacity of Alix binding [21]. Recently, we demonstrated that ALG-2 (a longer isoform, wild type) functions as a Ca^2+^-dependent adaptor protein that bridges Alix and TSG101, whereas ALG-2^ΔGF122^, retaining dimerization ability, does not have such a function toward Alix and TSG101 [38]. Occurrence of ALG-2^ΔGF122 ^in the cell should give chances to form dimers in different combinations at different ratios according to the expressed levels of wild-type ALG-2 and ALG-2^ΔGF122 ^(WT/WT, WT/ΔGF122, ΔGF122/ΔGF122). Although ALG-2 dimers of WT/ΔGF122 and ΔGF122/ΔGF122 are inactive adaptors at least for the Alix-type ALG-2-interacting proteins, the WT/ΔGF122 dimer may still function for non-Alix-type proteins. Although the molecular mechanism of the ALG-2 function in staurosporine-induced cell death is not known yet, slight augmentation of staurosporine-induced cell death by ALG-2^ΔGF122 ^(Figure 5) suggests that non-Alix type ALG-2-interacting proteins are also involved. Recent clinical investigations suggest that ALG-2 is a potential prognostic marker of certain lung and gastric cancers [39,40]. Expression analyses of ALG-2 by further distinguishing alternatively spliced isoforms would provide more reliable data in clinical studies. RBM22, a highly conserved RNA-binding protein functioning as an auxiliary factor of the spliceosome, was shown to interact with ALG-2 [41]. It would be interesting to see whether ALG-2 regulates its own splicing as well as Ca^2+^-dependent alternative splicing events [42]. Future studies are needed to clarify whether ALG-2^ΔGF122 ^plays roles merely as a negative inhibitor of wild-type ALG-2 or positively functions by associating with ALG-2^ΔGF122^-specific interacting proteins.

## Conclusions

Structural basis of the inability of the splicing isoform of human ALG-2, ALG-2^ΔGF122^, to bind to Alix was investigated by X-ray crystal structural analysis. Missing of two residues, Gly^121^Phe^122^, causes shortening of an α-helix (α5) and leads to a change in the configuration of the R125 side chain that resembles that of the metal-free form of ALG-2. Contrary to the expected importance of bulky side chain of F122, the F122A mutant exhibited a surprising hyperactivity in binding to Alix. The resolved structure of the F122A mutant showed that removal of the bulky F122 side chain not only created an additional open space in Pocket 2 but also abolished inter-helix interactions with W95 and V98 (present in α4) and that α5 inclined away from α4 to expand Pocket 2, suggesting acquirement of more appropriate positioning of the interacting residues to accept Alix. However, no hyperactivity against TSG101 or annexin A11 suggests that F122 partly restricts the recognition specificity to target proteins.

## Methods

### Bacterial expression and purification of recombinant ALG-2 proteins

Bacterial expressions of untagged ALG-2 by the T7 RNA polymerase system and GST-fused protein by pGEX vector were described previously [25,43]. Construction of the expression plasmid for an alternatively spliced ALG-2 isoform that lacks Gly^121^Phe^122 ^(designated ALG-2^ΔGF122^) and GST-ALG-2^ΔGF122 ^was described previously [25]. Single amino acid substitution mutation of ALG-2 was performed according to the instruction manual provided with a QuikChange Site-Directed Mutagenesis kit (Stratagene, USA) using specific oligonucleotide primers listed in Additional file 2, Table S4. Recombinant ALG-2 proteins of wild type and mutants (ALG-2, ALG-2^ΔGF122^, ALG-2^F122A^, ALG-2^F122G^, ALG-2^F122S^, ALG-2^F122W^, des3-23ALG-2^ΔGF122^, des3-20ALG-2^F122A^) were purified by affinity chromatography using a column immobilizing an ALG-2-binding site-2 (ABS-2) peptide of PLSCR3 as described previously [27]. GST fusion proteins were expressed and purified with glutathione Sepharose beads (GE Healthcare) according to the manufacturer's instructions.

### Crystallization

Purified proteins were concentrated to about 10 mg/ml with a vacuum centrifuge evaporator (Sakuma, Japan). Concentrated proteins were dialyzed against 10 mM Tris-HCl, pH 7.5, containing 10 μM each of EDTA and EGTA. Crystallization conditions were first screened with an automated robotic system [44] and further optimized manually. Crystals were grown by the sitting or hanging drop vapor diffusion method at 20°C. Des3-23ALG-2^ΔGF122 ^protein was crystallized with 25% (w/v) PEG-4000, 50 mM sodium cacodylate, pH 6.0, 300 mM ammonium acetate, and 10 mM calcium chloride. Des3-20ALG-2^F122A ^protein was crystallized with 25% (w/v) 2-methyl-2,4-pentanediol (MPD), 100 mM sodium cacodylate, pH 6.5, and 50 mM zinc acetate.

### Data collection, structure determination, refinement, and analyses

X-ray diffraction data were collected at beamlines BL-5A and NW-12 of Photon Factory (Tsukuba, Japan) under cryogenic conditions with crystals soaked in a cryoprotectant solution containing 20% glycerol and cooled to 100 K in a nitrogen gas stream. The diffraction data were integrated and scaled with the HKL2000 program package [45]. Crystal structures were solved by the molecular replacement method using the program MOLREP [46] with the published structure of ALG-2 (PDB ID 1HQV and 2ZN8) as a search model for des3-23ALG-2^ΔGF122 ^and des3-20ALG-2^F122A^. All models were refined with the programs CNS [47] and REFMAC5 in the CCP4 package [48]. Manual adjustments of the model were performed with COOT [49]. All of the structural figures were generated with PyMol (DeLano Scientific LLC, Palo Alto, CA). Rmsd was calculated with the program lsqkab in the CCP4 package [48]. Inter-helix angles and distances of EF-hand motifs were estimated by using vector geometry mapping (VGM) software [50] downloaded from URL: http://calcium.uhnres.utoronto.ca/vgm/.

### Binding assays

Real-time binding analyses were performed using an SPR biosensor (Biacore2000, GE Healthcare) at 25°C. A synthetic peptide of the ALG-2-binding site in Alix (kggsggsQGPPYPTYPGYPGYSQ, lower case residues indicating a linker, provided by Biosynthesis Inc., USA) was immobilized on the carboxymethylated dextran surface of a CM5 sensor chip (GE Healthcare) as described previously [21]. For interaction analyses, flow rate was maintained at 20 μl/min. Purified ALG-2 and mutants were diluted to 100 nM in HBS-P (10 mM HEPES-NaOH, pH 7.4, 150 mM NaCl, 0.005% surfactant P20) containing 100 μM CaCl_2 _and then injected and kept flowing over the immobilized sensor surface for 180 s. The sensor surface was then washed for 300 s with the same buffer and regenerated with the buffer containing 1 mM EGTA.

GST-pulldown assays of ALG-2 and its mutants were performed using cleared lysates of HEK293 cells as described previously [40]. Proteins bound to the beads (pulldown products) were analyzed by Western blotting using specific antibodies. Mouse monoclonal antibody (mAb) of anti-annexin VII (A-1) and goat polyclonal antibody (pAb) of anti-annexin XI (N-17) were obtained from Santa Cruz Biotechnology (Santa Cruz, CA). Anti-TSG101 mAb (4A10) and Sec31A mAb (clone32) were from Gene Tex (San Antonio, TX) and BD Transduction Laboratories (San Diego, CA), respectively. Preparation of anti-Alix pAb was described previously [25]. Signals of Western blotting were detected by the chemiluminescence method and analyzed with LAS-3000mini (Fujifilm, Japan).

### Cell culture, DNA transfection and cell death assays

Constructs of pcDNA3/ALG-2/RNAi^R ^and pcDNA3/ALG-2^ΔGF122^/RNAi^R ^that express RNAi-resistant ALG-2 mRNAs were obtained by transferring *Eco*RI/*Xho*I fragments to the pcDNA3 vector from their previously constructed expression vectors of FLAG-tagged versions [37]. Construction of pcDNA3/ALG-2^F122A^/RNAi^R ^was performed by site-directed mutagenesis using pcDNA3/ALG-2/RNAi^R ^as a template and the primers (F122A) listed in Additional file 2, Table S4. An ALG-2 knockdown HeLa cell line was established by expression of the short hairpin RNA specific for ALG-2 mRNA as described previously [25]. One day after the cells had been seeded, they were transfected with the expression plasmid DNAs by using FuGENE6 (Roche, Basel, Switzerland). After 24 h, aliquots of cell suspensions (each 0.5 ml) were transferred to a 24-well plate, incubated for 24 h, and then treated with or without 1 μM staurosporine for 24 h. Cell mortality was measured by quantifying the amount of lactate dehydrogenase (LDH) released from dead cells using the CytoTox96^® ^Non-Radioactive Cytotoxicity Assay (Promega, USA) according to the manufacturer's instructions. Total amount of LDH per sample (released and retained enzymes in dead and viable cells) was measured by lysing cells in 0.5 ml culture by adding the provided 10× Lysis buffer containing 9% Triton X-100.

## List of abbreviations

ABS: ALG-2-binding site; LDH: lactate dehydrogenase; PEF: penta-EF-hand; RNAi: RNA interference; SPR: surface plasmon resonance; TSG101: tumor susceptibility gene 101; WT: wild type

## Authors' contributions

TI and HSu carried out mutant preparations, purification of recombinant proteins, X-ray analyses of crystals. TI designed and performed cell death assays. MK and SW contributed in acquisition of X-diffraction data and interpretations of obtained data sets. TI, HSh and MM were involved in drafting and revising the manuscript. MM supervised the project. All authors read and approved the final version of the manuscript.

## Supplementary Material

Additional file 1 **Supplementary figures. **Showing structures of the calcium-bound dimeric form of des3-23ALG-2^ΔGF122 ^(Figure S1), the metal-free form of des3-20ALG-2 (Figure S2), EF-hand Ca^2+^-coordination in des3-23ALG-2^ΔGF122 ^(Figure S3), non-canonical Zn^2+^-coordination in EF5 (Figure S4), and SPR analyses of F122 mutants of ALG-2 for Alix-binding capacities (Figure S5). Click here for file

Additional file 2 **Supplementary tables.** Showing bond distances for the canonical EF-hand metal ion coordinates in ALG-2 for calcium (Table S1) and zinc (Table S2), bond distance for the non-canonical zinc ion coordinate in EF5 (Table S3), and primers used for site-directed mutagenesis performed in this study (Table S4). Click here for file

## References

[B1] Maki M Kitaura Y Satoh H Ohkouchi S Shibata H Structures, functions and molecular evolution of the penta-EF-hand Ca^2+^-binding proteins Biochim Biophys Acta 2002 1600 51 60 12445459 10.1016/s1570-9639(02)00444-2

[B2] Vito P Lacanà E D'Adamio L Interfering with apoptosis: Ca^2+^-binding protein ALG-2 and Alzheimer's disease gene ALG-3 Science 1996 271 521 525 10.1126/science.271.5248.521 8560270

[B3] Jang IK Hu R Lacanà E D'Adamio L Gu H Apoptosis-linked gene 2-deficient mice exhibit normal T-cell development and function Mol Cell Biol 2002 22 4094 4100 10.1128/MCB.22.12.4094-4100.2002 12024023 PMC133871

[B4] Rao RV Poksay KS Castro-Obregon S Schilling B Row RH del Rio G Gibson BW Ellerby HM Bredesen DE Molecular components of a cell death pathway activated by endoplasmic reticulum stress J Biol Chem 2004 279 177 187 10.1074/jbc.M304490200 14561754

[B5] la Cour JM Høj BR Mollerup J Simon R Sauter G Berchtold MW The apoptosis linked gene ALG-2 is dysregulated in tumors of various origin and contributes to cancer cell viability Mol Oncol 2008 1 431 439 10.1016/j.molonc.2007.08.002 19383317 PMC5543837

[B6] Høj BR la Cour JM Mollerup J Berchtold MW ALG-2 knockdown in HeLa cells results in G2/M cell cycle phase accumulation and cell death Biochem Biophys Res Commun 2009 378 145 148 19013425 10.1016/j.bbrc.2008.11.021

[B7] Missotten M Nichols A Rieger K Sadoul R Alix, a novel mouse protein undergoing calcium-dependent interaction with the apoptosis-linked-gene 2 (ALG-2) protein Cell Death Differ 1999 6 124 129 10.1038/sj.cdd.4400456 10200558

[B8] Vito P Pellegrini L Guiet C D'Adamio L Cloning of AIP1, a novel protein that associates with the apoptosis-linked gene ALG-2 in a Ca^2+^-dependent reaction J Biol Chem 1999 274 1533 1540 10.1074/jbc.274.3.1533 9880530

[B9] Odorizzi G The multiple personalities of Alix J Cell Sci 2006 119 3025 3032 10.1242/jcs.03072 16868030

[B10] Morita E Sandrin V Chung HY Morham SG Gygi SP Rodesch CK Sundquist WI Human ESCRT and ALIX proteins interact with proteins of the midbody and function in cytokinesis EMBO J 2007 26 4215 4227 10.1038/sj.emboj.7601850 17853893 PMC2230844

[B11] Carlton JG Martin-Serrano J The ESCRT machinery: new functions in viral and cellular biology Biochem Soc Trans 2009 37 195 199 10.1042/BST0370195 19143630

[B12] Raiborg C Stenmark H The ESCRT machinery in endosomal sorting of ubiquitylated membrane proteins Nature 2009 458 445 452 10.1038/nature07961 19325624

[B13] Schmidt MH Hoeller D Yu J Furnari FB Cavenee WK Dikic I Bögler O Alix/AIP1 antagonizes epidermal growth factor receptor downregulation by the Cbl-SETA/CIN85 complex Mol Cell Biol 2004 24 8981 893 10.1128/MCB.24.20.8981-8993.2004 15456872 PMC517880

[B14] Pan S Wang R Zhou X Corvera J Kloc M Sifers R Gallick GE Lin SH Kuang J Extracellular Alix regulates integrin-mediated cell adhesions and extracellular matrix assembly EMBO J 2008 27 2077 2090 10.1038/emboj.2008.134 18636094 PMC2516883

[B15] Mahul-Mellier AL Strappazzon F Petiot A Chatellard-Causse C Torch S Blot B Freeman K Kuhn L Garin J Verna JM Fraboulet S Sadoul R Alix and ALG-2 are involved in tumor necrosis factor receptor 1-induced cell death J Biol Chem 2008 283 34954 34965 10.1074/jbc.M803140200 18936101 PMC3259881

[B16] Maki M Narayana SV Hitomi K A growing family of the Ca^2+^-binding proteins with five EF-hand motifs Biochem J 1997 328 718 720 9441591 PMC1218977

[B17] Jia J Han Q Borregaard N Lollike K Cygler M Crystal structure of human grancalcin, a member of the penta-EF-hand protein family J Mol Biol 2000 300 1271 1281 10.1006/jmbi.2000.3925 10903868

[B18] Jia J Tarabykina S Hansen C Berchtold M Cygler M Structure of apoptosis-linked protein ALG-2: insights into Ca^2+^-induced changes in penta-EF-hand proteins Structure 2001 9 267 275 10.1016/S0969-2126(01)00585-8 11525164

[B19] Xie X Dwyer MD Swenson L Parker MH Botfield MC Crystal structure of calcium-free human sorcin: a member of the penta-EF-hand protein family Protein Sci 2001 10 2419 2425 11714909 10.1110/ps.36701 PMC2374028

[B20] Ilari A Johnson KA Nastopoulos V Verzili D Zamparelli C Colotti G Tsernoglou D Chiancone E The crystal structure of the sorcin calcium binding domain provides a model of Ca^2+^-dependent processes in the full-length protein J Mol Biol 2002 29 447 458 10.1006/jmbi.2002.5417 11922676

[B21] Suzuki H Kawasaki M Inuzuka T Okumura M Kakiuchi T Shibata H Wakatsuki S Maki M Structural basis for Ca^2+^-dependent formation of ALG-2/Alix peptide complex: Ca^2+^/EF3-driven arginine switch mechanism Structure 2008 16 1562 1573 10.1016/j.str.2008.07.012 18940611

[B22] Suzuki H Kawasaki M Inuzuka T Okumura M Kakiuchi T Shibata H Wakatsuki S Maki M The mechanism of Ca^2+^-dependent recognition of Alix by ALG-2: insights from X-ray crystal structures Biochem Soc Trans 2009 37 190 194 10.1042/BST0370190 19143629

[B23] Crivici A Ikura M Molecular and structural basis of target recognition by calmodulin Ann Rev Biophys Biomol Struct 1995 24 85 116 10.1146/annurev.bb.24.060195.000505 7663132

[B24] Tarabykina S Møller AL Durussel I Cox J Berchtold MW Two forms of the apoptosis-linked protein ALG-2 with different Ca^2+ ^affinities and target recognition J Biol Chem 2000 275 10514 10518 10.1074/jbc.275.14.10514 10744743

[B25] Katoh K Suzuki H Terasawa Y Mizuno T Yasuda J Shibata H Maki M The penta-EF-hand protein ALG-2 interacts directly with the ESCRT-I component TSG101, and Ca^2+^-dependently co-localizes to aberrant endosomes with dominant-negative AAA ATPase SKD1/Vps4B Biochem J 2005 391 677 685 10.1042/BJ20050398 16004603 PMC1276969

[B26] Shibata H Suzuki H Yoshida H Maki M ALG-2 directly binds Sec31A and localizes at endoplasmic reticulum exit sites in a Ca^2+^-dependent manner Biochem Biophys Res Commun 2007 353 756 763 10.1016/j.bbrc.2006.12.101 17196169

[B27] Shibata H Suzuki H Kakiuchi T Inuzuka T Yoshida H Mizuno T Maki M Identification of Alix-type and Non-Alix-type ALG-2-binding sites in human phospholipid scramblase 3: differential binding to an alternatively spliced isoform and amino acid-substituted mutants J Biol Chem 2008 283 9623 9632 10.1074/jbc.M800717200 18256029

[B28] Nakano H Omura S Chemical biology of natural indolocarbazole products: 30 years since the discovery of staurosporine J Antibiot 2009 62 17 26 10.1038/ja.2008.4 19132059

[B29] Nicolier M Decrion-Barthod AZ Launay S Prétet JL Mougin C Spatiotemporal activation of caspase-dependent and -independent pathways in staurosporine-induced apoptosis of p53wt and p53mt human cervical carcinoma cells Biol Cell 2009 101 455 467 10.1042/BC20080164 19216720

[B30] Subramanian L Crabb JW Cox J Durussel I Walker TM van Ginkel PR Bhattacharya S Dellaria JM Palczewski K Polans AS Ca^2+ ^binding to EF hands 1 and 3 is essential for the interaction of apoptosis-linked gene-2 with Alix/AIP1 in ocular melanoma Biochemistry 2004 43 11175 11186 10.1021/bi048848d 15366927 PMC1351334

[B31] Shibata H Yamada K Mizuno T Yorikawa C Takahashi H Satoh H Kitaura Y Maki M The penta-EF-hand protein ALG-2 interacts with a region containing PxY repeats in Alix/AIP1, which is required for the subcellular punctate distribution of the amino-terminal truncation form of Alix/AIP1 J Biochem 2004 135 117 128 10.1093/jb/mvh014 14999017

[B32] Yap KL Ames JB Swindells MB Ikura M Diversity of conformational states and changes within the EF-hand protein superfamily Proteins 1999 37 499 507 10.1002/(SICI)1097-0134(19991115)37:3<499::AID-PROT17>3.0.CO;2-Y 10591109

[B33] Suzuki H Kawasaki M Kakiuchi T Shibata H Wakatsuki S Maki M Crystallization and X-ray diffraction analysis of N-terminally truncated human ALG-2 Acta Crystallogr Sect F Struct Biol Cryst Commun 2008 64 974 977 10.1107/S1744309108030297 18997320 PMC2581688

[B34] Maki M Shibata H The Penta-EF-hand protein ALG-2 and its interacting proteins. Calcium Binding Proteins Calcium Binding Proteins 2007 2 4 10

[B35] Vergarajauregui S Martina JA Puertollano R Identification of the penta-EF-hand protein ALG-2 as a Ca^2+^-dependent interactor of mucolipin-1 J Biol Chem 2009 284 36357 36366 10.1074/jbc.M109.047241 19864416 PMC2794751

[B36] Hwang IS Jung YS Kim E Interaction of ALG-2 with ASK1 influences ASK1 localization and subsequent JNK activation FEBS Lett 2002 529 183 187 10.1016/S0014-5793(02)03329-X 12372597

[B37] Chen C Sytkowski AJ Apoptosis-linked gene-2 connects the Raf-1 and ASK1 signalings Biochem Biophys Res Commun 2005 333 51 57 10.1016/j.bbrc.2005.05.074 15925322

[B38] Okumura M Ichioka F Kobayashi R Suzuki H Yoshida H Shibata H Maki M Penta-EF-hand protein ALG-2 functions as a Ca^2+^-dependent adaptor that bridges Alix and TSG101 Biochem Biophys Res Commun 2009 386 237 241 10.1016/j.bbrc.2009.06.015 19520058

[B39] Yamada Y Arao T Gotoda T Taniguchi H Oda I Shirao K Shimada Y Hamaguchi T Kato K Hamano T Koizumi F Tamura T Saito D Shimoda T Saka M Fukagawa T Katai H Sano T Sasako M Nishio K Identification of prognostic biomarkers in gastric cancer using endoscopic biopsy samples Cancer Sci 2008 99 2193 2199 10.1111/j.1349-7006.2008.00935.x 18957060 PMC11158963

[B40] Aviel-Ronen S Coe BP Lau SK da Cunha Santos G Zhu CQ Strumpf D Jurisica I Lam WL Tsao MS Genomic markers for malignant progression in pulmonary adenocarcinoma with bronchioloalveolar features Proc Natl Acad Sci USA 2008 105 10155 10160 10.1073/pnas.0709618105 18632575 PMC2465804

[B41] Montaville P Dai Y Cheung CY Giller K Becker S Michalak M Webb SE Miller AL Krebs J Nuclear translocation of the calcium-binding protein ALG-2 induced by the RNA-binding protein RBM22 Biochim Biophys Acta 2006 1763 1335 1343 10.1016/j.bbamcr.2006.09.003 17045351

[B42] Krebs J The influence of calcium signaling on the regulation of alternative splicing Biochim Biophys Acta 2009 1793 979 984 10.1016/j.bbamcr.2008.12.006 19133299

[B43] Satoh H Nakano Y Shibata H Maki M The penta-EF-hand domain of ALG-2 interacts with amino-terminal domains of both annexin VII and annexin XI in a Ca^2+^-dependent manner Biochim Biophys Acta 2002 1600 61 67 12445460 10.1016/s1570-9639(02)00445-4

[B44] Hiraki M Kato R Nagai M Satoh T Hirano S Ihara K Kudo N Nagae M Kobayashi M Inoue M Uejima T Oda S Chavas LM Akutsu M Yamada Y Kawasaki M Matsugaki N Igarashi N Suzuki M Wakatsuki S Development of an automated large-scale protein-crystallization and monitoring system for high-throughput protein-structure analyses Acta Crystallogr D Biol Crystallogr 2006 62 1058 1065 10.1107/S0907444906023821 16929107

[B45] Otwinowski Z Minor W Processing of X-ray diffraction data collected in oscillation mode Methods Enzymol 1997 276 307 326 full_text 10.1016/S0076-6879(97)76066-X 27754618

[B46] Vagin A Teplyakov A MOLREP: an automated program for molecular replacement J Appl Crystallogr 1997 30 1022 1025 10.1107/S0021889897006766

[B47] Brünger AT Adams PD Clore GM DeLano WL Gros P Grosse-Kunstleve RW Jiang JS Kuszewski J Nilges M Pannu NS Read RJ Rice LM Simonson T Warren GL Crystallography & NMR system: A new software suite for macromolecular structure determination Acta Crystallogr D Biol Crystallogr 1998 54 905 921 10.1107/S0907444998003254 9757107

[B48] Murshudov GN Vagin AA Dodson E Refinement of macromolecular structures by the maximum-likelihood method Acta Crystallogr D Biol Crystallogr 1997 53 240 255 10.1107/S0907444996012255 15299926

[B49] Emsley P Cowtan K Coot: model-building tools for molecular graphics Acta Crystallogr D Biol Crystallogr 2004 60 2126 2132 10.1107/S0907444904019158 15572765

[B50] Yap KL Ames JB Swindells MB Ikura M Vector geometry mapping. A method to characterize the conformation of helix-loop-helix calcium-binding proteins Methods Mol Biol 2002 173 317 324 11859772 10.1385/1-59259-184-1:317

