# Supplementary material for: Molecular basis for defect in Alix-binding by alternatively spliced isoform of ALG-2 (ALG-2ΔGF122) and structural roles of F122 in target recognition
Source: BMC Struct Biol. 2010 Aug 6;10:25. doi: 10.1186/1472-6807-10-25 (PMC2927601; doi:10.1186/1472-6807-10-25)
Supplement: Additional file 1 — Supplementary figures. Showing structures of the calcium-bound dimeric form of des3-23ALG-2ΔGF122 (Figure S1), the metal-free form of des3-20ALG-2 (Figure S2), EF-hand Ca2+-coordination in des3-23ALG-2ΔGF122 (Figure S3), non-canonical Zn2+-coordination in EF5 (Figure S4), and SPR analyses of F122 mutants of ALG-2 for Alix-binding capacities (Figure S5). [file 1472-6807-10-25-S1.PDF]

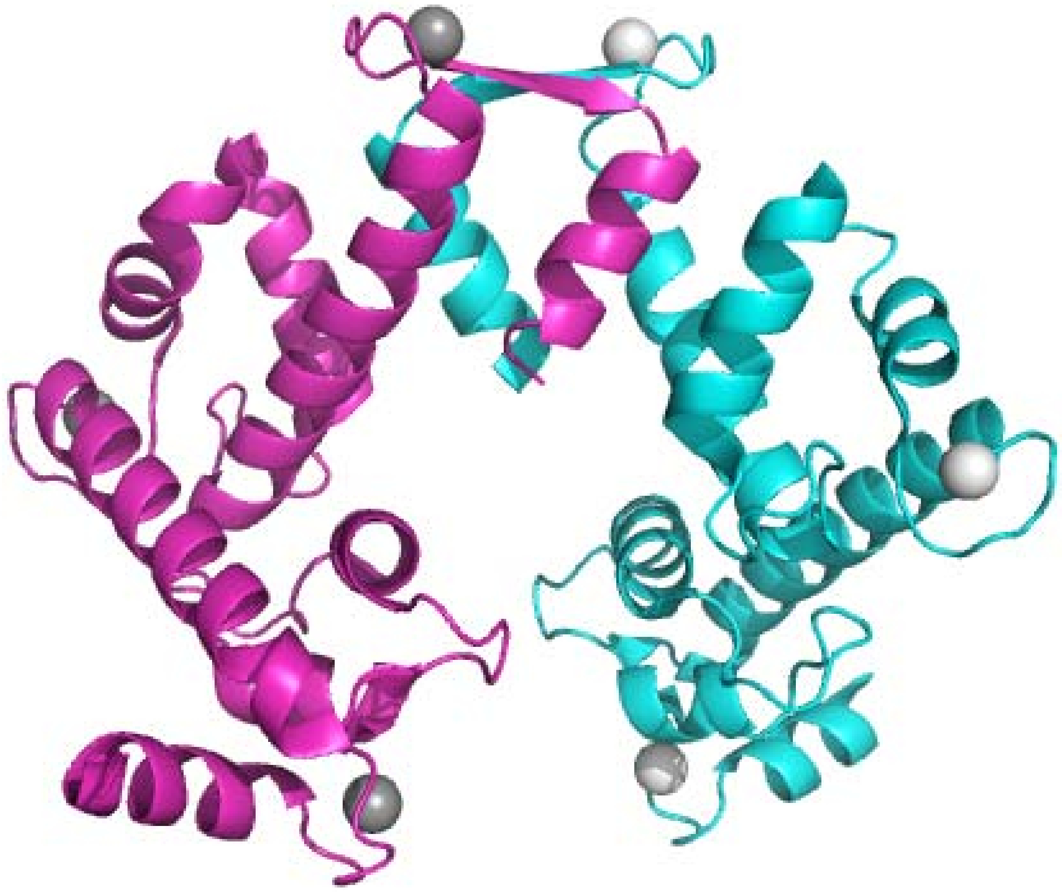

Figure S1

Structures of the dimer form of des3-23ALG-2$^{\Delta GF122}$.

Two monomers (molecules A and B) are shown in magenta and cyan in ribbon representation and EF-hand-coordinated calcium ions are shown as gray and white spheres, respectively. The structures aligned between molecules A and B have a root-mean-square deviation (rmsd) value of 0.73 Å for C^{α} atoms from residues Leu28 to Val189

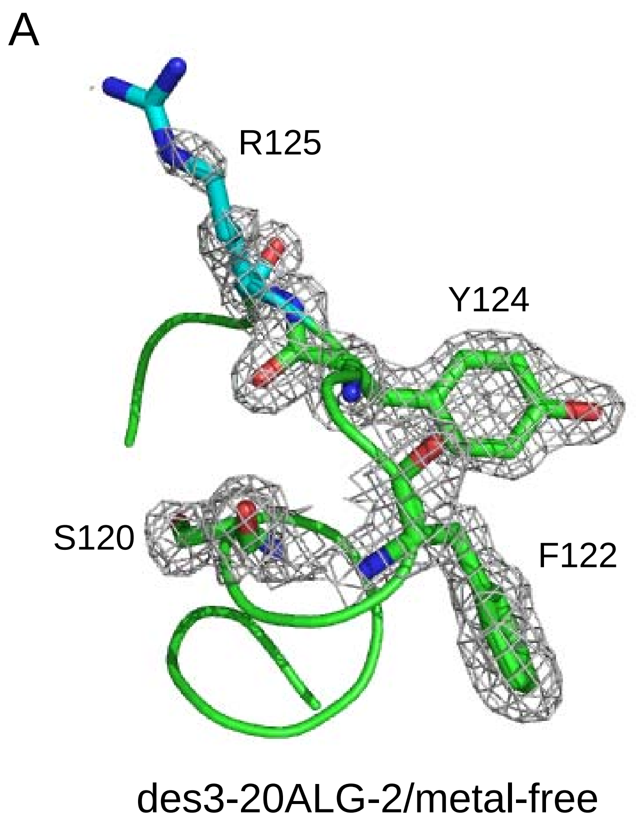

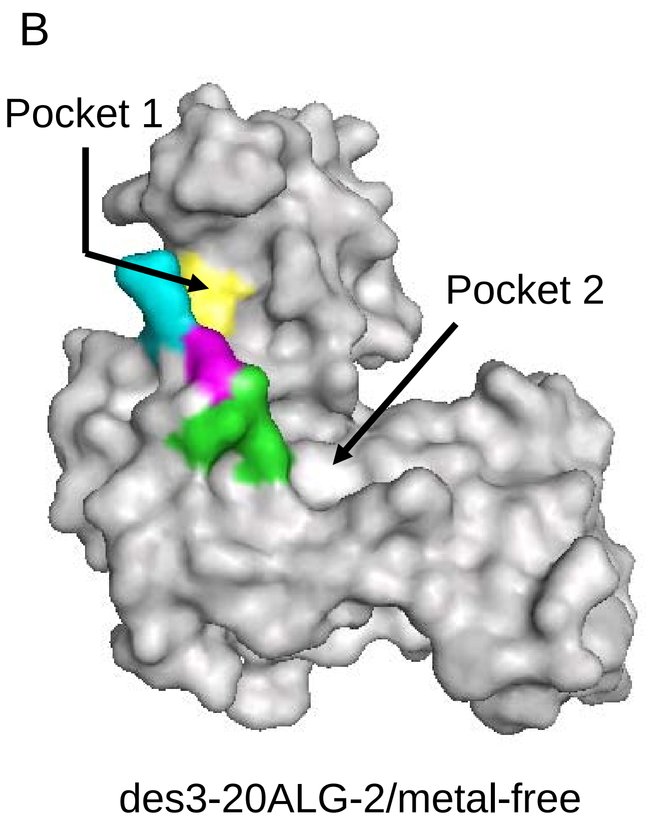

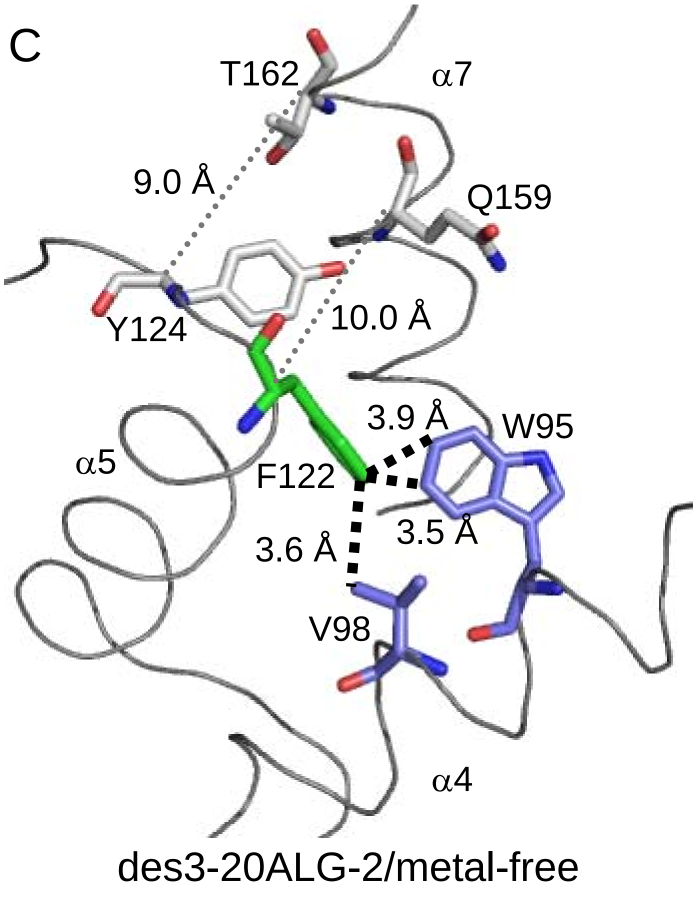

Figure S2

Structures of the metal-free form of des3-20ALG-2. (A) Configuration of the R125 side chain as shown in Figure 2. (B) Surface structure as shown in Figure 3. (C) Close-up view of structural interrelations among α4, α5 and α7 as shown in Figure 6.

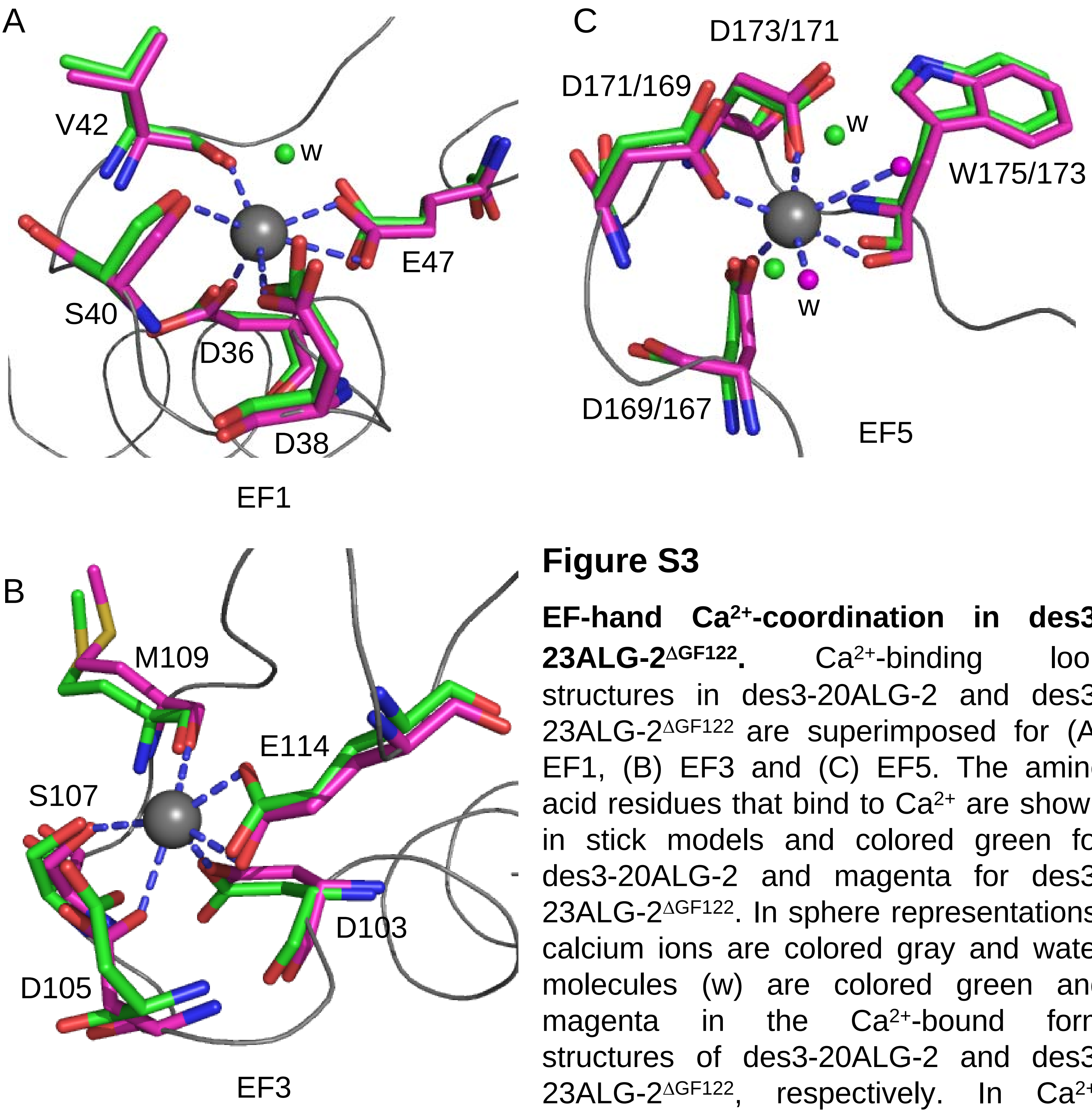

Figure S3

EF-hand Ca^{2+}-coordination in des3-23ALG-2$^{\Delta GF122}$. Ca^{2+}-binding loop structures in des3-20ALG-2 and des3-23ALG-2$^{\Delta GF122}$ are superimposed for (A) EF1, (B) EF3 and (C) EF5. The amino acid residues that bind to Ca^{2+} are shown in stick models and colored green for des3-20ALG-2 and magenta for des3-23ALG-2$^{\Delta GF122}$. In sphere representations, calcium ions are colored gray and water molecules (w) are colored green and magenta in the Ca^{2+}-bound form structures of des3-20ALG-2 and des3-23ALG-2$^{\Delta GF122}$, respectively. In Ca^{2+}-bound des3-23ALG-2$^{\Delta GF122}$, the main chain is represented by a ribbon, and bonds between Ca^{2+} and oxygen atoms of either amino acid residues or water molecules are indicated by light blue bars.

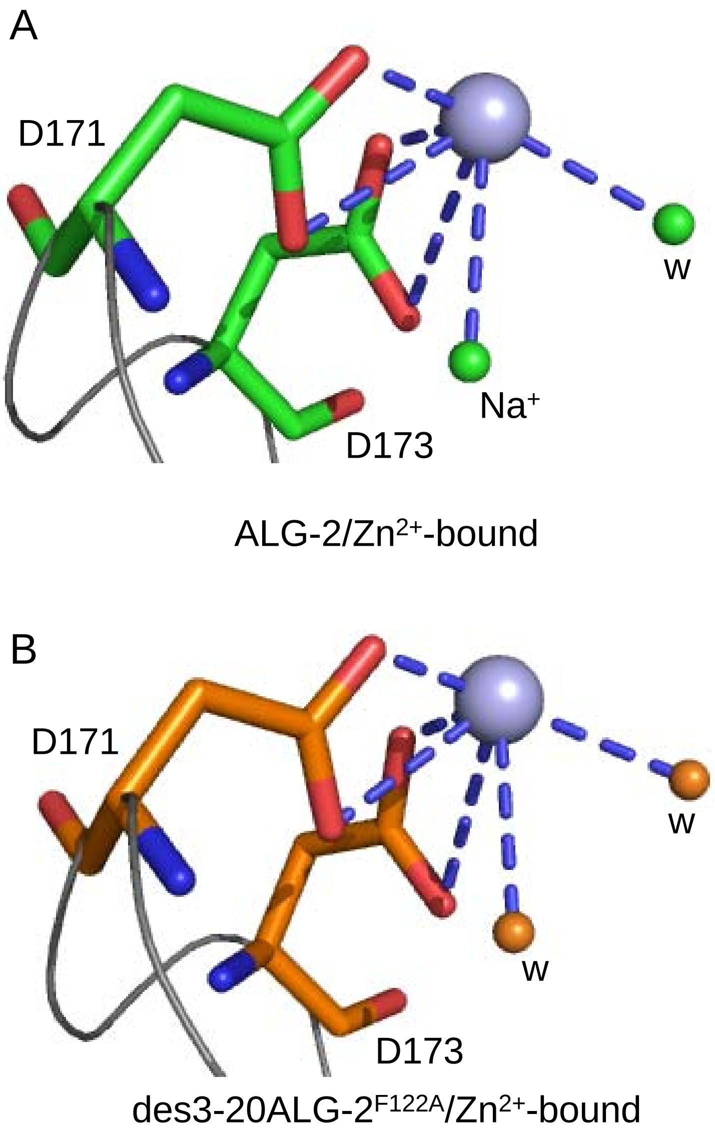

Figure S4

Non-canonical Zn^{2+}-coordination in EF5. In Zn^{2+}-bound (A) ALG-2 and (B) des3-20ALG-2^{F122A}, the amino acid residues coordinating Zn^{2+} are shown in stick models and colored green and orange, respectively. In sphere representations, zinc ions are colored light cyan and a sodium ion (Na^{+}) and a water molecule (w) are colored green (A) or orange (B) as indicated. Light blue bars represent bonds between Zn^{2+} and oxygen atoms of the concerned residues or water molecules.

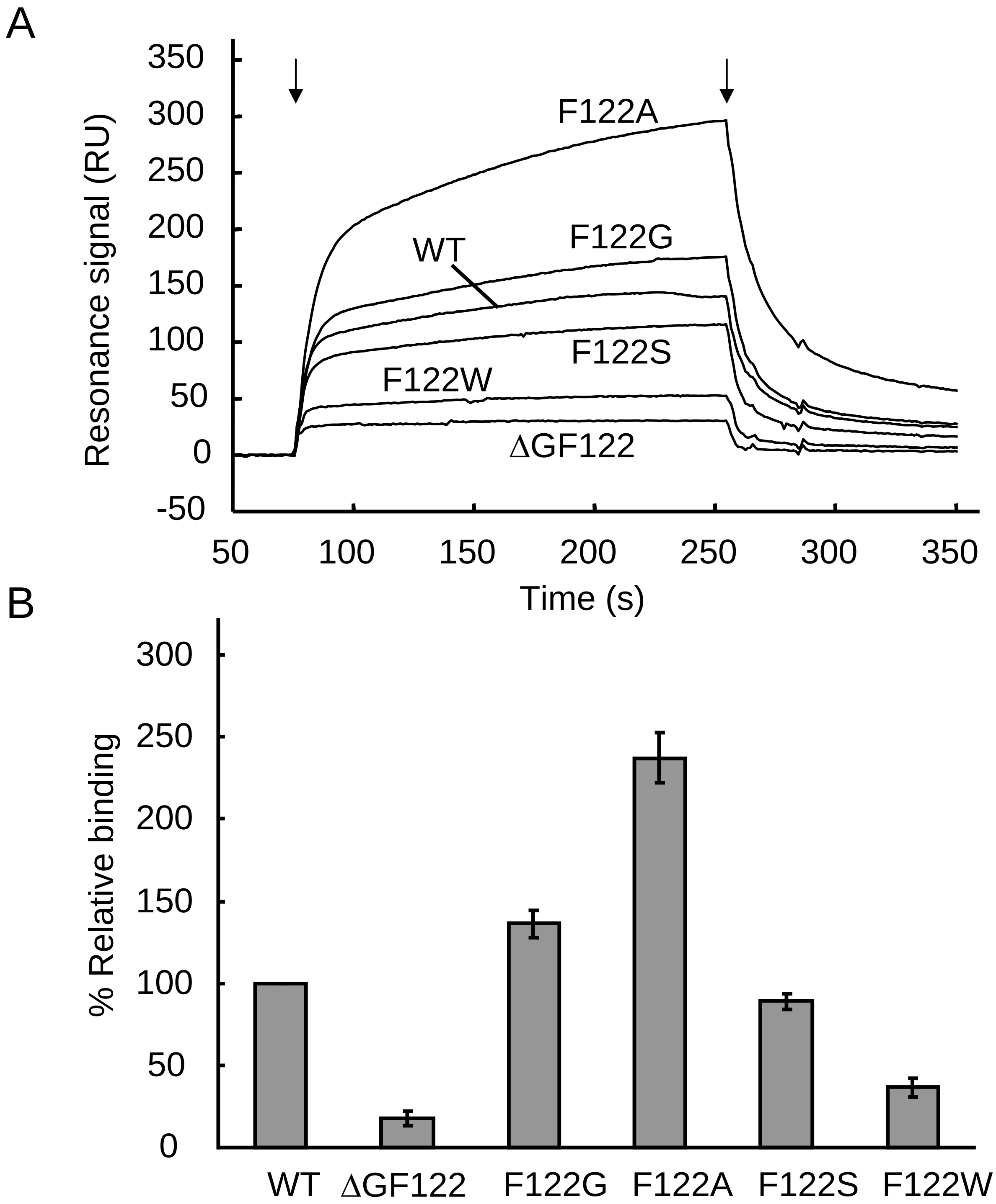

Figure S5

SPR analyses of F122 mutants of ALG-2 for Alix-binding capacities. (A) Real-time interaction analyses using an SPR biosensor were performed by injecting a solution of 100 nM each of ALG-2 or mutants in a running buffer containing 100 μM $CaCl_2$. Resonance signals from the flow cell where an ALG-2-binding site (ABS) peptide of Alix was immobilized were subtracted by those in a reference flow cell whose sensor chip surface was blocked by ethanolamine. Arrows indicate injection time of analyte (at 75 s) and buffer (at 255 s). (B) Relative binding capacity was calculated by expressing the resonance units (RU) of the ALG-2 sample (WT) at 10 s before the end of injection (at 245 s) as 100%. All data are presented as means +/- SD (n = 7).
