# Supplementary material for: Molecular basis for defect in Alix-binding by alternatively spliced isoform of ALG-2 (ALG-2ΔGF122) and structural roles of F122 in target recognition
Source: BMC Struct Biol. 2010 Aug 6;10:25. doi: 10.1186/1472-6807-10-25 (PMC2927601; doi:10.1186/1472-6807-10-25)
Supplement: Additional file 2 — Supplementary tables. Showing bond distances for the canonical EF-hand metal ion coordinates in ALG-2 for calcium (Table S1) and zinc (Table S2), bond distance for the non-canonical zinc ion coordinate in EF5 (Table S3), and primers used for site-directed mutagenesis performed in this study (Table S4). [file 1472-6807-10-25-S2.PDF]

Table S1: Bond distance for the calcium ion coordinate in ALG-2

	EF1			EF3			EF5		
		Distance (Å)			Distance (Å)			Distance (Å)	
Position	Atom	WT[a]	ΔGF122[b]	Atom	WT	ΔGF122	Atom	WT	ΔGF122
x	$OD1^{D36}$	2.3	2.2	$OD1^{D103}$	2.3	2.3	$OD1^{D169}$	2.5	2.3
y	$OD2^{D38}$	2.2	2.4	$OD2^{D105}$	2.5	2.5	$OD2^{D171}$	2.3	2.4
z	OG^{S40}	2.8	2.5	OG^{S107}	2.5	2.6	$OD1^{D173}$	2.2	2.4
-y	O^{V42}	2.4	2.3	O^{M109}	2.4	2.5	O^{W175}	2.5	2.4
-x	H_2O	2.3	ND[c]	H_2O	ND	ND	H_2O	2.3	2.8
-z	$OE1^{E47}$	2.8	2.5	$OE1^{E114}$	2.3	2.5	H_2O	2.4	2.2
	$OE2^{E47}$	2.6	2.5	$OE2^{E114}$	2.5	2.5			

[a] Ca^{2+}-bound form of des3-20ALG-2
[b] Ca^{2+}-bound form of des3-23ALG-$2^{\Delta GF122}$
[c] Not detected

Table S2: Bond distance for the zinc ion coordinate in ALG-2

	EF1			EF3			EF5		
		Distance (Å)			Distance (Å)			Distance (Å)	
Position	Atom	WT[a]	F122A[b]	Atom	WT	F122A	Atom	WT	F122A
x	OD1^{D36}	2.2	3.2	OD1^{D103}	2.3	2.0	OD1^{D169}	ND[c]	ND
y	OD2^{D38}	2.3	2.1	OD2^{D105}	2.4	2.1	OD2^{D171}	ND	ND
z	OG^{S40}	2.5	2.9	OG^{S107}	2.8	2.1	OD1^{D173}	ND	ND
-y	O^{V42}	2.1	2.2	O^{M109}	2.5	3.0	O^{W175}	ND	ND
-x	H_2O	ND	2.2	H_2O	2.8	2.5	H_2O	ND	ND
-z	OE1^{E47}	2.5	2.6	OE1^{E114}	2.4	2.5	H_2O	ND	ND
	OE2^{E47}	2.8	2.8	OE2^{E114}	2.8	3.0			

[a] Zn^{2+}-bound form of ALG-2
[b] Zn^{2+}-bound form of des3-20ALG-2^{F122A}
[c] Not detected

Table S3: Bond distance for the non-canonical zinc ion coordinate in EF5 of ALG-2

	EF5 Distance (Å)	
Atom	WT[a]	F122A[b]
OD1[D171]	2.9	2.8
OD2[D171]	2.0	1.9
OD1[D173]	3.1	3.1
OD2[D173]	2.0	1.8
H_2O	2.6	2.6
Na/H_2O	3.0	3.0

[a] Zn^{2+}-bound form of ALG-2
[b] Zn^{2+}-bound form of des3-20ALG-2[F122A]

Table S4: Primers used for site-directed mutagenesis performed in this study

Mutation	Orientation	Nucleotide sequence
F122G	Forward	5'-ccctctcaggtggcggctaccggctc-3'
	Reverse	5'-gagccggtagccgccacctgagaggg-3'
F122A	Forward	5'-gccctctcaggtgccggctaccggc-3'
	Reverse	5'-gccggtagccggcacctgagagggc-3'
F122S	Forward	5'-gccctctcaggtagcggctaccggctc-3'
	Reverse	5'-gagccggtagccgctacctgagagggc-3'
F122W	Forward	5'-gccctctcaggttggggctaccggctc-3'
	Reverse	5'-gagccggtagccccaacctgagagggc-3'
